# CALENDAR

**DOI:** 10.1038/sj.bjc.6690212

**Published:** 1999-03


					11-13 April 1999
MICRO 2000 International Microscopy Exhibition and
Conference
London, UK
Further information from:
Alison Winton, Exhibition Organizer, Royal Microscopical
Society, 37/38 St Clements, Oxford OX4 1AJ, UK.
E-mail: exhibitions@rms.org.uk
16 April 1999
Joint Meeting of the Section of Urology, Royal Society
of Medicine and the British Prostate Group - Prostate
Cancer: The Impact of Basic Science on Screening,
Treatment and Quality of Life
London, UK
Further information from:
Meeting Secretariat, c/o British Association of Urological
Surgeons, 35/43 LincolnOs Inn Fields, London WC2A 3PN, UK.
Tel: +44 (0) 171 405 1390, Fax: +44 (0) 171 404 5048
17-19 May 1999
Radiology 1999-Imaging, Science & Oncology
International Convention Centre, Birmingham, UK
Proferred papers are invited for poster presentation. Deadline for
Work in Progress: 22 February 1999
Further information from:
Radiology 1999 Secretariat, 36 Portland Place, London W1N 4AT,
UK. Tel: +44 (0) 171 307 1410, Fax: +44 (0) 171 307 1414
Erratum
Monoclonal antibodies directed to the rb-2 receptor
inhibit in vivo tumor cell growth
I-M Harwerth, W Wels, J Schlegel and NE Hynes
Br J Cancer 68: 1140-1185
In the interest of scientific accuracy and our mutual desire with
the editors that only high quality and reliable data are published,
we regret to say that the in vivo data for the article above are not
considered reliable. A technician with responsibility for
conducting in vivo tumor xenograft experiments admitted to
manipulating data for some of the experiments he performed. We
wish to retract the data until new in vivo experiments have been
repeated and reevaluated. The validity of the in vitro and cellular
data is not in doubt. We very much regret this situation.
CALENDAR
1318
British Journal of Cancer (1999) 79(7/8), 1318
(c) 1999 Cancer Research Campaign
Article no. bjoc.1998.0212

				

